# Comparison of clinical and dermoscopic features between extramammary Paget’s disease and chronic eczema

**DOI:** 10.3389/fmed.2026.1791704

**Published:** 2026-03-24

**Authors:** Lingjian Wu, Liyue Hou, Junyi Shao, Enci Mei, Jingjing Liu

**Affiliations:** First Affiliated Hospital of Wenzhou Medical University, Wenzhou, China

**Keywords:** chronic eczema, clinical differentiation, dermoscopy, diagnosis, extramammary Paget’s disease

## Abstract

Extramammary Paget’s disease (EMPD) is a rare cutaneous malignancy that is easily confused with other diseases, especially chronic eczema. This study focuses on the clinical and dermoscopy characteristics of EMPD and chronic eczema, aiming to identify the key points of differentiation between the two and evaluate the role of dermoscopy in differential diagnosis. The study retrospectively analyzed 40 patients with EMPD and 40 patients with chronic eczema who were diagnosed with histopathological examination in the dermatology department of our hospital from January 2020 to December 2024. The clinical and dermoscopy characteristics of the two groups were analyzed, and differences were compared using *t*-tests and chi-square tests. *P* < 0.01 was considered statistically significant. Clinically, chronic eczema group had more common itching and significantly increased scales; while EMPD patients had a higher incidence of nodules, pigmentation, and hypopigmentation. Dermatoscopy revealed that the background color of EMPD lesions was mainly milky red, with a vascular pattern of evenly distributed punctate and globular vessels; chronic eczema was characterized by clustered punctate vessels, with statistically significant differences (*P* < 0.01). In addition, the EMPD group had a higher frequency of gray/brown dots, gray/brown amorphous areas, bright white lines, bright white amorphous areas, and reticular structures, with statistically significant differences (*P* < 0.01). This study is the first to conduct a comparative analysis on a large sample size of 80 cases. The results indicate that EMPD and chronic eczema differ in clinical and dermoscopy characteristics, and dermoscopy plays a significant role in the early and accurate diagnosis of EMPD.

## Introduction

Extramammary Paget disease (EMPD) is a malignant tumor originating from apocrine glands (1, 2). It usually affects people aged 60–80, most commonly postmenopausal white women and Asian men (3). EMPD often occurs in areas rich in apocrine glands outside the breast, such as the perianal area, scrotum, penis, vulva region of female and axilla (4). The disease progresses slowly, and the skin lesions appear as eczematous changes, presenting as well-defined, infiltrative erythema, which may be accompanied by erosion and exudation (1). Due to its low malignancy, it is often misdiagnosed as eczema for a long time in clinical practice. Histopathological examination is the main basis for the diagnosis of EMPD, but it is an invasive procedure. Dermoscopy is a non-invasive method and has shown certain advantages in the early screening of skin tumors (5). Many studies have reported and summarized the dermoscopic manifestations of EMPD (6), but there are few reports comparing the dermoscopic features of EMPD and chronic eczema with similar clinical manifestations. This study aims to preliminarily observe the clinical manifestations and dermoscopic characteristics of the two and explore the value of dermoscopy in their differential diagnosis.

## Related works

We have previously described the dermoscopic characteristics of EMPD (2). In this study, we collected cases of EMPD and eczema in the perineum area and analyzed the dermoscopic characteristics.

## Methodology

A retrospective analysis was performed to compare the dermoscopic characteristics of the two groups. Chi-square test and t test were used for statistical analysis using spss27 software. *P* < 0.01 was regarded as statistically significant.

## Experimental setup

A retrospective study was conducted on 40 patients with extramammary Paget’s disease (EMPD) and 40 patients with chronic eczema who consecutively visited the dermatology department of our hospital from January 2020 to December 2024. This study was approved by the Medical Ethics Committee of our Hospital. All patients underwent histopathological examination for a confirmed diagnosis. Inclusion criteria as follows: (1) Lesions located in the perineum area; (2) Pathologically confirmed as EMPD or chronic eczema; (3) Complete clinical and dermoscopic data. Images were collected and acquired using dermoscopy (Dermoscopy Image Processing Workstation DERMOSCOPY-II, Wenzhou Hemei Medical Instrument Co., Ltd. polarized light source, X20/X50, Suzhou Guo Ke Ying Rui Medical Technology Co., Ltd. X20). Dermoscopic images of the lesions to be pathologically examined were collected, with 3 images per patient. All dermoscopic images were collected by the same dermatologist. Dermoscopic diagnoses were independently made by two dermatologists trained in dermoscopy.

The following dermoscopic features were determined based on relevant literature (2, 6, 7): (1) Background color: milky-red areas, light red areas; (2) surface scales: white scales, yellow scales; (3) vascular morphology: dotted vessels, glomerular vessels; (4) vascular distribution: uniform distribution and clustered distribution; (5) linear irregular vessels; (6) gray/brown dots; (7) gray/brown structured areas; (8) shiny white line; (9) white structureless area; (10) reticular structure: including “chrysalis-like structures” which is described as shiny white line paralleled or intersectinged and lava lake structure which is defined as a combination of branching white reticular lines (6, 7). Therefore, we use “reticulate structure” to describe these characteristics. To protect patient privacy and confidentiality, multiple measures were implemented. Modern imaging systems with AI-assisted recognition were used to automatically detect and blur sensitive anatomical regions such as the chest and genital areas, with local mosaic processing or encrypted storage applied immediately after image generation. Strict access control was enforced through the Picture Archiving and Communication System (PACS), where only attending physicians or higher-level personnel could apply to unlock and view the images when clinically necessary, and all access was logged for traceability. Additionally, image data was transmitted within the hospital via a dedicated secure network to prevent leakage, and when provided externally, anonymization was performed to remove patient identifying information.

For continuous variables (e.g., age, disease duration), *t*-tests were employed, with normality assumptions rigorously assessed via the Shapiro-Wilk test (all *P* > 0.05, indicating no significant deviation from normality). For categorical variables (e.g., sex, vascular features), chi-square tests were conducted, ensuring all expected frequencies exceeded 5 in every cell to maintain test validity. All statistical analyses were conducted using SPSS version 27.0. The *t*-test and chi-square test were employed, with *P* < 0.01 indicating statistical significance.

## Results and discussion

### Clinical features

**FIGURE 1:**
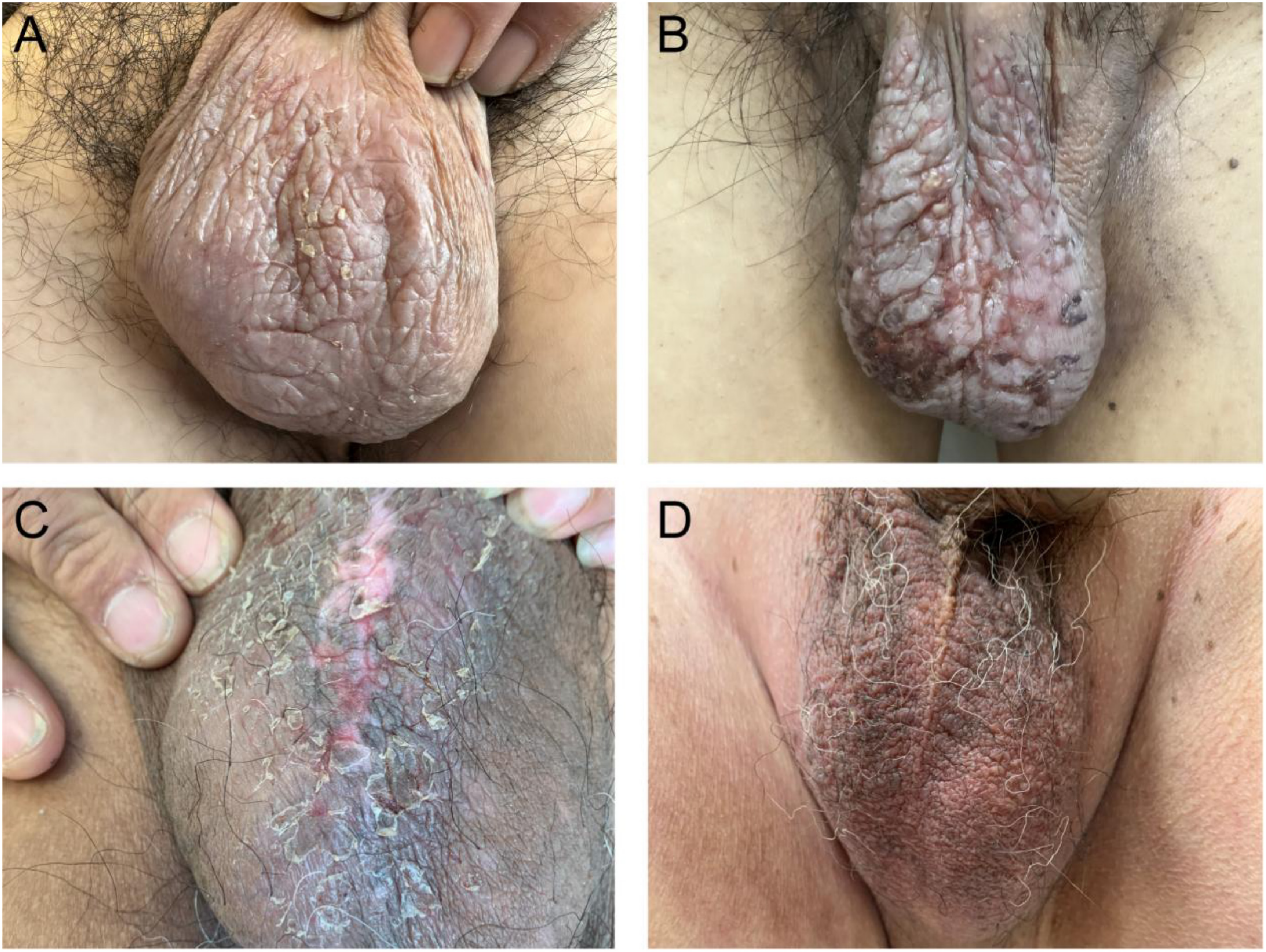
Clinical features of chronic eczema. **(A)** Light red plaques with lichenification, crusting and slight scaling. **(B)** Red plaques with lichenification, partial ulceration and crusting. **(C)** Dark red plaques with lichenification, crusting and scaling. **(D)** Red plaques with slight lichenification.

**FIGURE 2:**
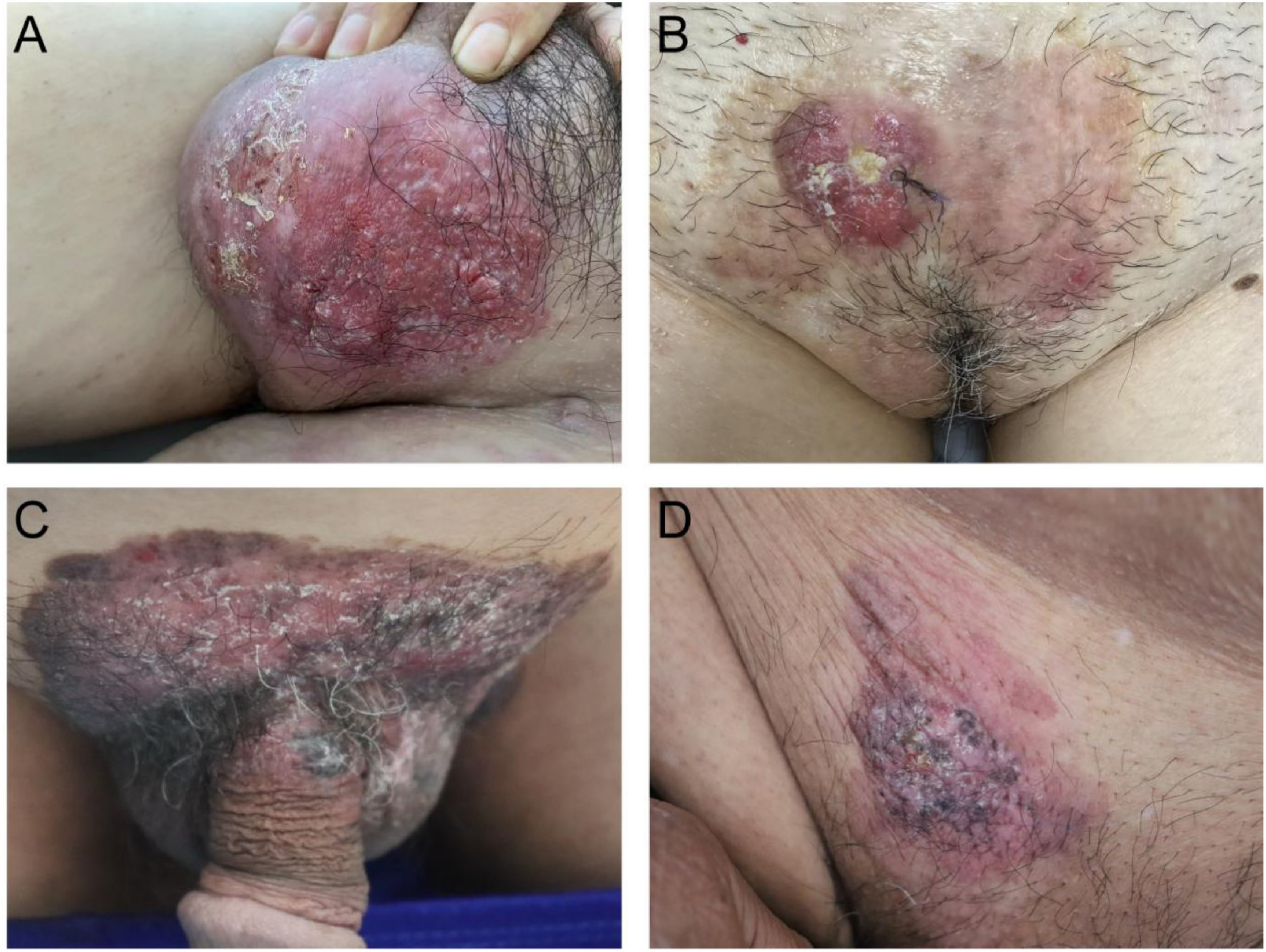
**(A)** Red plaques on the scrotal area associated with localized nodular hyperplasia and hypopigmented patches. **(B)** Red plaques on the female mons pubis, accompanied by localized ulceration and crusting. **(C)** Dark red plaques on the mons pubis and scrotal area, accompanied by pigmentation and scaling. **(D)** Red plaques on the male mons pubis, accompanied by pigmentation.

The Clinical Features for each group are summarized in Tables 1, 2 and illustrated in Figures 1, 2.

**TABLE 1:** Basic information.

Variable		Group		*t*/χ^2^	*p*
		EMPD	Eczema		
Gender	Male	35	30	2.051^b^	0.152
	Female	5	10		
Age		69.55 ± 9.17	48.30 ± 12.33	8.745^a^	0.000**
Course of disease		3.50 ± 2.01	1.45 ± 1.27	5.442^a^	0.000**
Location	Scrotum	21	18	10.365^b^	0.035*
	Penis	4	7		
	Scrotum + penis	3	1		
	Mons pubis	12	7		
	Lateral labia majora	0	7		

*p < 0.05, **p < 0.01, ^a^*t*-test, ^b^Chi-square test.

**TABLE 2:** Clinical features.

Variable		Group		*t*/χ^2^	*P*
		EMPD	eczema		
Color of lesions	Erythema/plaques	26	20	1.841^b^	0.175
	Light erythema/plaques	14	20		
Itch	No	29	4	32.237^b^	0.000**
	Yes	11	36		
Scales	No	28	12	12.800^b^	0.000**
	Yes	12	28		
Exudation	No	27	36	6.050^b^	0.014*
	Yes	13	4		
Nodules	No	27	40	15.522^b^	0.000**
	Yes	13	0		
Pigmentation	No	19	34	12.579^b^	0.000**
	yes	21	6		
Hypopigmentation	no	17	39	28.810^b^	0.000**
	yes	23	1		

*p < 0.05, **p < 0.01, ^b^Chi-square test.

A total of 40 patients with EMPD and 40 patients with chronic eczema were included in the study. The mean ages were 69.55 ± 9.17 years and 48.30 ± 12.33 years, respectively. The male-to-female ratios were 7:1 in the EMPD group and 3:1 in the eczema group. The mean disease duration was 3.50 ± 2.01 years in the EMPD group and 1.45 ± 1.27 years in the eczema group. In elderly individuals, the degradation of skin barrier function and increased fragility of microvessels may exacerbate the neoplastic vascular proliferation characteristics of EMPD (such as dot-and-ball-shaped vessels), while the inflammatory vascular reactions of eczema (such as patchy erythema) are more common in younger populations. The decline in estrogen levels may indirectly shape the typical vascular pattern of EMPD by affecting the skin barrier and vascular reactivity; whereas male patients with eczema may present atypical lesions (such as lichenification) due to hormonal fluctuations, which can interfere with diagnosis. The most commonly affected site in both groups was the scrotum, with 21 cases in the EMPD group and 18 cases in the eczema group. Clinically, both groups presented with erythema or plaques, sometimes accompanied by scales and crusts. Pruritus and scaling were significantly more common in the eczema group than in the EMPD group (*P* < 0.01). If age and gender were not adjusted, the observed differences in vascular characteristics in the study (*P* < 0.001) may be partly attributed to baseline population structural differences rather than inherent disease differences, thereby exerting a systematic influence on the interpretation of results. On the one hand, punctate-globular vessels may be mistakenly considered as “exclusive” features of EMPD, when in fact they may represent skin aging manifestations in elderly women, leading to an overestimation of diagnostic specificity. On the other hand, atypical lesions in young EMPD patients or male eczema patients may be excluded from the “typical pattern,” posing a risk of missed diagnosis and underestimating diagnostic sensitivity. Furthermore, attributing “age/gender-related vascular changes” to the pathological process of EMPD can confound causal inference and weaken the validity of dermoscopy as an independent diagnostic tool. This study is a single-center retrospective design without multivariable adjustment, and it does not report the independent effects of age and gender on vascular characteristics (such as odds ratio or beta coefficient), nor does it conduct stratified analysis (such as by age < 60 years vs. ≥ 60 years, or gender grouping), limiting the internal validity of the conclusions and making it impossible to distinguish between “disease-specific” and “population-characteristic” manifestations.

Nodules were observed in 13 patients with EMPD but in none of the eczema patients. Pigmentation was present in 21 cases of EMPD and 6 cases of eczema, while hypopigmentation was observed in 23 cases of EMPD and only 1 case of eczema. The differences in the presence of nodules, pigmentation, and hypopigmentation between the two groups were statistically significant (*P* < 0.01).

### Dermoscopic features

**FIGURE 3:**
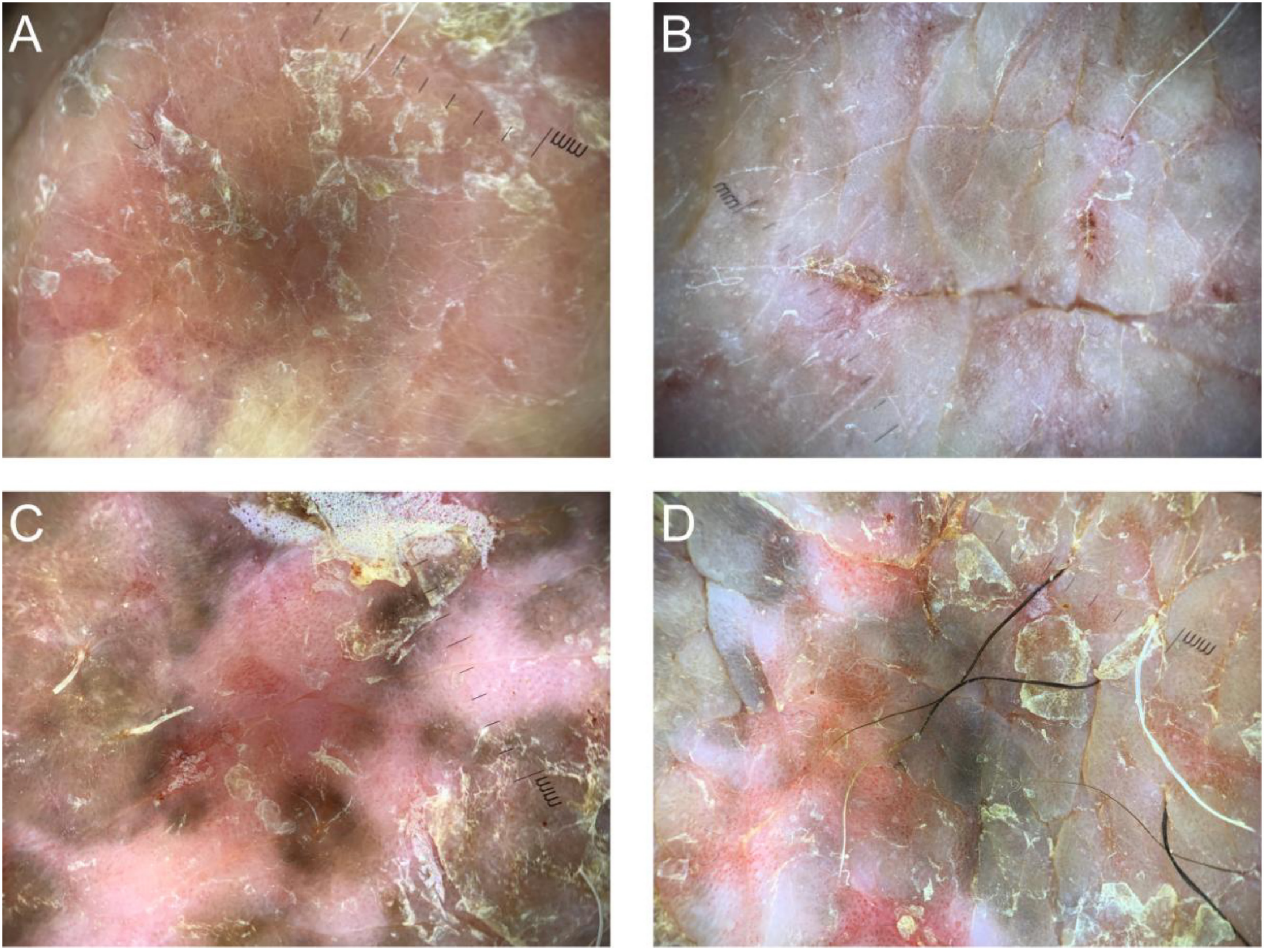
Dermoscopic features of chronic eczema. **(A)** A light red background with linear vessels and scaling. **(B)** A light red background with cluster-like distribution of dotted vessels and a small amount of crusting. **(C,D)** A light red background with cluster-like distribution of dotted vessels and hyperpigmentation and scales.

**FIGURE 4:**
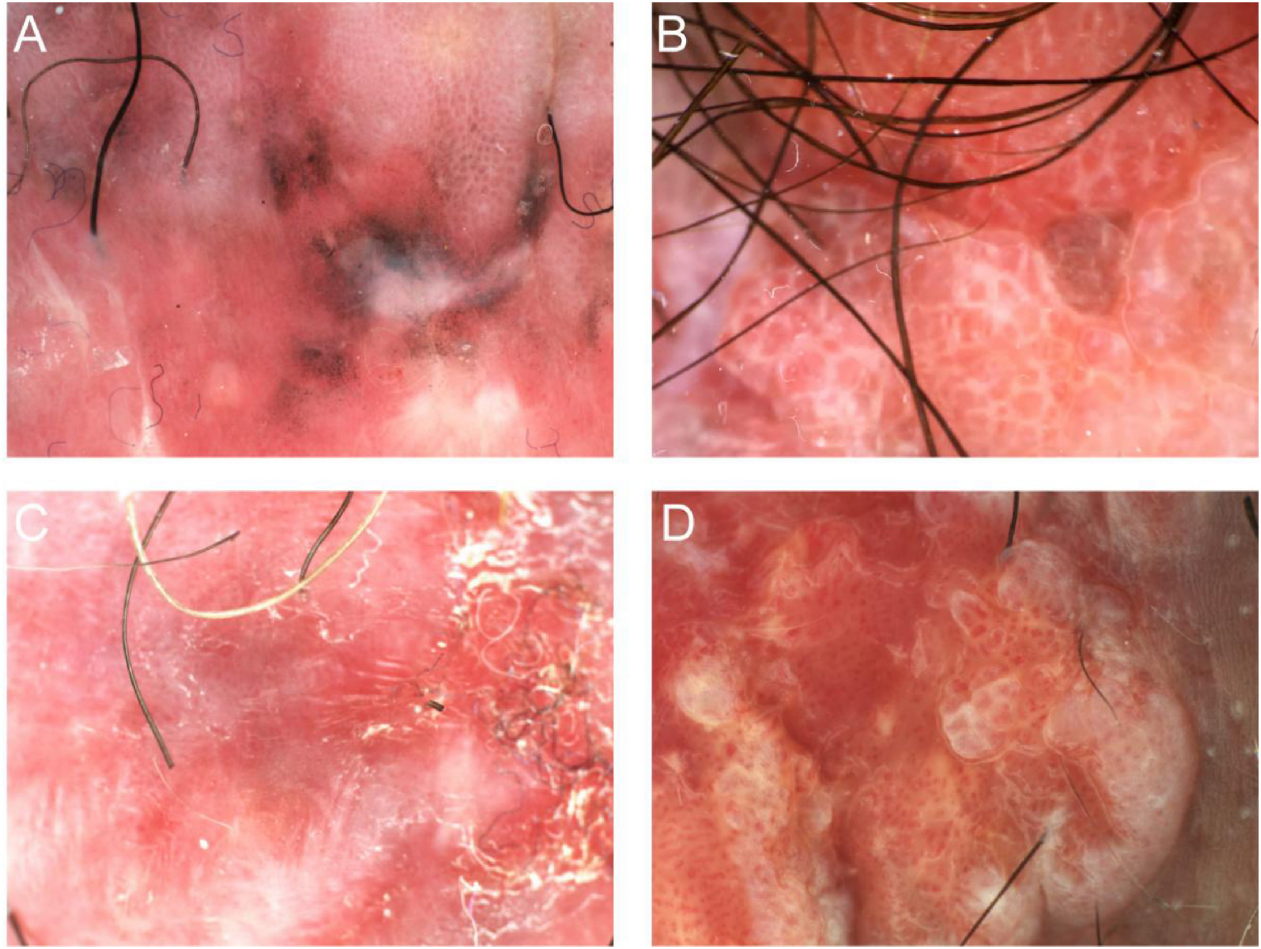
Dermoscopic features of EMPD. **(A)** A milky-red background with gray/brown dots, gray/brown areas, reticular structure, and white structureless areas. **(B)** A milky-red background with a reticular structure. **(C)** A milky-red background with shiny white line and white structureless areas. **(D)** A milky-red background with diffuse dotted vessels and glomerular vessels.

The dermoscopic findings for each group are summarized in Tables 3, 4 and illustrated in Figures 3, 4. A milky-red background was observed in all 40 cases of EMPD, whereas a pale milky-red background was noted in 30 cases of chronic eczema, with a statistically significant difference between the two groups (*P* < 0.01). The presence of scales was significantly more common in the EMPD group.

**TABLE 3:** Dermoscopic features.

Variable		Group		χ^2^	*P*
		EMPD	Eczema		
Milky-red areas	No	0	10	11.429	0.001**
	Yes	40	30		
Surface scales	No scaling	23	7	21.003	0.000**
	White scales	9	29		
	Yellow scales	4	3		
	White scales + yellow scales	4	1		

**p < 0.01.

**TABLE 4:** Typical dermoscopic features.

variable		Group		χ^2^	*p*
		EMPD	Eczema		
Dotted vessels	No	0	23	32.281	0.000**
	Yes	40	17		
Glomerular vessels	No	5	30	31.746	0.000**
	Yes	35	10		
Linear irregular vessels	No	40	30	11.429	0.001**
	Yes	0	10		
Gray/brown dots	No	21	39	21.600	0.000**
	Yes	19	1		
Grey/brown structured areas	No	19	38	22.029	0.000**
	Yes	21	2		
Shiny white line	No	4	39	61.596	0.000**
	Yes	36	1		
White structureless area	No	0	38	72.381	0.000**
	Yes	40	2		
Reticular structure	No	13	40	40.755	0.000**
	Yes	27	0		

**p < 0.01.

In the EMPD group, 40 patients demonstrated a uniform vascular distribution, with 35 cases showing glomerular vessels, In contrast, in the eczema group, 11 cases exhibited patchy or clustered vascular patterns, 10 cases showed dotted-globular vessels. The differences in vascular characteristics and distribution between the two groups were statistically significant (*P* < 0.01). Additionally, linear vessels were observed in 10 cases in the eczema group, whereas none were found in the EMPD group.

In the EMPD group, 19 cases presented with gray/brown dots and 21 cases with gray/brown structured areas, compared to only 1 and 2 cases, respectively, in the eczema group (*P* < 0.01). Shiny white lines were observed in 36 EMPD cases and in only 1 eczema case, while white structureless areas were seen in 40 EMPD cases and 2 eczema cases, with all differences reaching statistical significance (*P* < 0.01). Additionally, a reticulate structure was identified in 27 cases of EMPD, but was absent in all eczema cases. In dermoscopy diagnosis, the characteristic differences between EMPD and chronic eczema have significant pathological bases: the EMPD group (40 cases) all exhibited a milky red background, correlating with neoplastic vascular proliferation, and its pathological mechanism may involve the arrangement of melanocyte nests or fibrotic collagen bundles at the epidermal-dermal junction; whereas 75% of the eczema group (30 cases) presented with a pale milky red background, reflecting inflammatory vascular reactions (such as punctate vascular clustering). In terms of vascular distribution patterns, 87.5% of the EMPD group exhibited uniform vascular distribution, predominantly glomerular-like vessels, indicating destructive infiltration of tumor cells into normal skin texture; whereas 55% of the eczema group showed patchy or clustered vessels, mainly punctate-globular vessels, reflecting the damage to the skin barrier by inflammatory reactions, and linear vessels were only observed in the eczema group (10 cases). Regarding pigmented lesion characteristics, 47.5% of the EMPD group presented with gray/brown spots, correlating with tumor cell pigmentation or local inflammatory reactions, whereas only 2.5% of the eczema group exhibited similar manifestations; the reticular structure was observed in 67.5% of the EMPD group, reflecting the arrangement of melanocyte nests or fibrotic collagen bundles at the epidermal-dermal junction, while this structure was absent in the eczema group. Additionally, bright white stripes were present in 90% of the EMPD group, correlating with periodic striations or elastin aggregation of collagen fibers in the dermis, whereas only 2.5% of the eczema group exhibited such manifestations; unstructured areas accounted for 52.5% of the EMPD group, correlating with disordered tumor cell arrangement or deep infiltration leading to structural disappearance, whereas only 5% of the eczema group had such areas. Comprehensive analysis of these characteristics aids in distinguishing neoplastic from inflammatory lesions, but histopathological confirmation is necessary.

EMPD is a rare intraepidermal adenocarcinoma first reported by Crocker in 1889 (8, 9). The incidence of this disease is relatively low. In 2012, the incidence of EMPD in Europe was approximately 0.7 per million and in the Chinese population, it was about 0.4 per million (9). It commonly occurs in areas with dense apocrine sweat glands, such as the scrotum, penis, and perianal region. Clinically, it often presents as red patches or plaques of varying sizes with clear boundaries. Some cases may present as nodules, erosions, ulcers, hyperplasia, hypopigmentation, or hyperpigmentation, and may be accompanied by symptoms such as itching (10). Due to its appearance similar to eczema, EMPD is also called “eczematoid carcinoma” and needs to be differentiated from eczema. Therefore, if patients have persistent eczema-like skin lesions in these areas, they should undergo histopathological examination for early diagnosis. Histopathological examination is the gold standard for diagnosing EMPD. Microscopically, it mainly shows pale vacuolated Paget cells appearing singly or in nests within the spinous layer. Paget cells are large and round, with large pale nuclei and disappearance of intercellular bridges (11). Given the invasive nature and high recurrence rate of EMPD, early diagnosis and intervention are essential (12).

In recent years, dermoscopy, reflectance confocal microscopy (RCM), and high-frequency ultrasonography have been widely used in the diagnosis of skin tumors and inflammatory dermatoses, with dermoscopy being especially favored for its non-invasive, convenient, and cost-effective advantages (5, 13–15). Previous literature has reported some dermoscopic features of EMPD (14), but there are few comparative reports between EMPD and chronic eczema. This study summarized the clinical manifestations and dermoscopic features of 40 cases of EMPD and 40 cases of chronic eczema. We found that in terms of clinical manifestations, Patients in the EMPD group exhibited a significantly older age at onset and a longer disease duration compared to the eczema group. Nevertheless, the eczema group was more likely to have itching, while the EMPD group was mainly asymptomatic. Eczema patients were more likely to have scales than EMPD patients, which may be related to scratching caused by itching. At the same time, EMPD patients were more likely to have nodules, hypopigmentation, and hyperpigmentation. Under dermoscopy, the background of EMPD was mostly diffuse milky red, while the eczema group had a light red background in some cases, which was similar to the observation of Mun et al. (14). Milky-red areas may be associated with tumor-related neoangiogenesis. White scales are the most common desquamation form in eczema, while EMPD shows white scales and yellow scales. The vascular structures in EMPD patients were consistently distributed, with more Dotted and Glomerular vessels; in contrast, the vascular structures in chronic eczema were patchy or clustered. EMPD patients were more likely to have gray/brown dots, gray/brown structured areas, shiny white line and white structureless area.

Additionally, the reticular structure was only observed in the EMPD group and not in the eczema group, which should be a characteristic structure of EMPD. In this study, a higher proportion of linear vessels was observed in patients with eczema, which may be related to the thin skin, rich and tortuous vascular network of the scrotal (or vulvar) area, or to previous treatment with topical corticosteroids.

This study is a single-center retrospective design. Despite the continuous inclusion of patients, there may still be selection bias. Firstly, single-center data may be limited by specific medical environments, patient population characteristics (such as geography, economy, and medical insurance policies), and doctors’ diagnostic and treatment preferences, which restricts the external generalizability of the results. Secondly, retrospective data rely on past records, which may have information gaps or recording biases, especially in non-standardized follow-up indicators. Although we have tried to control bias through strict inclusion/exclusion criteria and multi-source data cross-validation, we cannot completely exclude the influence of unmeasured confounding factors. Future multi-center prospective studies will help verify the universality of this result.

This study has several potential limitations: the sample size is limited and sourced from a single institution, potentially including only retrospective cases from a single medical institution, which may not be representative of a broader population, especially omitting asymptomatic or mildly symptomatic patients who did not seek medical attention, leading to selection bias; retrospective data are missing, and clinical and dermoscopy image data rely on previous medical records, which may result in the absence of key variables (such as previous history of topical medication, hormone use, time point of treatment intervention, itching scores, etc.), affecting the completeness of feature comparison; dermoscopy image acquisition standards are not uniform, and equipment, light source conditions, magnification, shooting angles, and operator techniques are not standardized, which may lead to systematic errors in feature interpretation and reduce the reproducibility of results; inter-observer variability is not quantified, and dermoscopy feature interpretation relies on subjective experience. If no inter-observer consistency test (such as Kappa value) is reported, the impact of inter-diagnostic differences on the results cannot be evaluated; there is a lack of comprehensive verification by pathological gold standards, and the pathological results may not fully match the dermoscopy features, resulting in an incomplete “gold standard” and affecting the evaluation of differential diagnostic efficacy; in addition, the study did not control for confounding variables (such as age, gender, duration of disease, comorbid dermatoses, etc.), which may confound the characteristic differences between EMPD and chronic eczema.

## Conclusion

In conclusion, this was our first comparative study with a relatively large number involving 80 cases. We found that there are some differences in clinical manifestations between EMPD and eczema. The dermoscopic manifestations of the EMPD group are more diverse and rich, which provides a certain basis for the early diagnosis of EMPD and can play a certain role in early diagnosis. However, the sample size of this study was limited, and we look forward to larger sample size data to provide more evidence in the future. This study verifies the regional applicability of dermoscopy features in the local Chinese population for the first time: This study includes patients from the Wenzhou region, filling the empirical gap due to differences in climate, skin characteristics, and disease manifestations among the local Chinese population, and enhancing the geographical universality of diagnostic criteria.

## Data Availability

The raw data supporting the conclusions of this article will be made available by the authors, without undue reservation.
